# False negativity for trisomy 18 in non-invasive prenatal testing: A case report

**DOI:** 10.1016/j.crwh.2026.e00813

**Published:** 2026-04-21

**Authors:** Natália Andová, Vanda Repiská, Petra Priščáková, Lajos Gergely, Michaela Hýblová, Gabriel Minárik

**Affiliations:** a Institute of Medical Biology, Genetics and Clinical Genetics, Faculty of Medicine, Comenius University Bratislava, Bratislava, Slovakia; b MEDIREX GROUP ACADEMY n.p.o., Nitra, Slovakia; c Trisomy test Ltd., Nitra, Slovakia

**Keywords:** Diagnostic errors, Mosaicism, Non-invasive prenatal testing, Trisomy 18 syndrome

## Abstract

False-negative results in non-invasive prenatal testing are rare but clinically significant, particularly in the presence of biological factors such as feto-placental mosaicism. This report describes a pregnancy affected by fetal trisomy 18 in which three consecutive non-invasive prenatal tests reported low risk despite abnormal first-trimester ultrasound findings (increased nuchal translucency, hydrops fetalis, and tricuspid regurgitation). Amniocentesis subsequently confirmed trisomy 18 in the fetus. Post-termination placental analysis revealed mosaicism, with high trisomy 18 levels on the fetal side (up to 76%) and lower levels on the maternal side (20%) of the placenta. This distribution resulted in an estimated “effective fetal fraction” below the standard detection threshold for a non-invasive prenatal test (∼4%). These findings highlight the limitations of non-invasive prenatal testing as a screening test and underscore the importance of invasive diagnostic testing when structural abnormalities are detected. Consideration of biological variables such as placental mosaicism is essential for accurate interpretation and clinical decision-making.

## Highlights

• Feto-placental mosaicism may lead to false-negative NIPT.
• Uneven placental mosaicism masked trisomy 18 in cfDNA.
• Repeated NIPT does not exclude false-negative results.
• Diagnostic testing remains essential with structural anomalies.

## 1 Introduction

Clinical practice is quickly adopting non-invasive prenatal testing (NIPT) based on next-generation sequencing of cell-free fetal DNA (cffDNA) fragments that can be found in maternal circulation [1]. cffDNA is known to originate from the fetal-placental unit and is a minor component of the maternal circulating DNA. Thus, vanishing twin syndrome, maternal malignancy, and feto-placental mosaicism are the primary known causes of NIPT's inaccuracy [2].

Currently, common fetal aneuploidies are detected through prenatal screening. Non-invasive prenatal tests are categorized as screening methods, and they provide a probability assessment regarding whether the unborn child has a specific genetic alteration. Ultimately, an invasive prenatal test is necessary to confirm the findings [3].

A meta-analysis by Taylor-Phillips et al. [4] revealed that NIPT for trisomy 18 (T18, Edwards syndrome) has a sensitivity of 97.4% and a specificity of 99.9%. These results closely align with findings for the Trisomy test® (*N* = 20,288, internal data), which demonstrated a sensitivity of 97.96% and a specificity of 99.99%. Similarly, a more recent meta-analysis by Liehr [3] reported sensitivity and specificity rates for T18 of 97.97% and 99.96%, respectively, further confirming that false-negative results for Edwards syndrome are rare. For genome-wide techniques, NIPT results for an individual are expressed as a *Z*-score, which refers to the number of standard deviations from the mean of a reference data set [5].

This report describes a fetus with abnormalities detected by ultrasound examination and T18 confirmed by diagnostic testing following false-negative NIPT results explained by feto-placental mosaicism in which the placenta (cytotrophoblast and/or mesenchyme) exhibited a mosaic of euploid and aneuploid cell lines, while the fetus contained only the aneuploid cell line [2].

## 2 Case Presentation

A 31-year-old healthy gravida with a singleton pregnancy was referred for NIPT at her personal request. At 12 + 2 weeks of gestation, the patient underwent the Trisomy Test® through the protocol published by Sekelska et al. [6]. The result was uninformative, with *Z*-scores for T21 = 2.74, T18 = 2.61, and T13 = 3.09 at the fetal fraction of 9.66%. The Z-scores were all within the “grey zone” (2.5–4), where it is not possible to clearly determine whether the test result is high risk or low risk [7]. Repeat NIPT performed at 13 + 3 weeks produced a low-risk result, with *Z*-scores for T21 = 1.61, T18 = 0.81, and T13 = 1.07 at the fetal fraction of 7.64%. Despite these reassuring findings, first-trimester ultrasonography revealed multiple fetal anomalies: pathologic nuchal translucency (NT) thickness of 5.35 mm, hydrops fetalis, and tricuspid regurgitation, all features commonly associated with chromosomal abnormalities [8], [9].

Given the concerning ultrasonographic findings, an amniocentesis was performed at 18 + 3 weeks of gestation and analysed using array comparative genomic hybridization (aCGH) to assess the entire genome of a patient for genetic alterations. The analysis of the patient's native amniotic fluid sample revealed a duplication of 77.878 Mbp corresponding to the entire chromosome 18. This result is consistent with a diagnosis of Edwards syndrome.

Additionally, a third non-invasive prenatal test, at 21 + 6 weeks of gestation, was performed, again with low-risk results, at the fetal fraction of 10.66% and *Z*-scores T21 = 2.08, T18 = 0.31, and T13 = −0.28. Following comprehensive genetic counselling and based on the definitive results from the invasive test, the pregnancy was terminated.

**Fig. 1:**
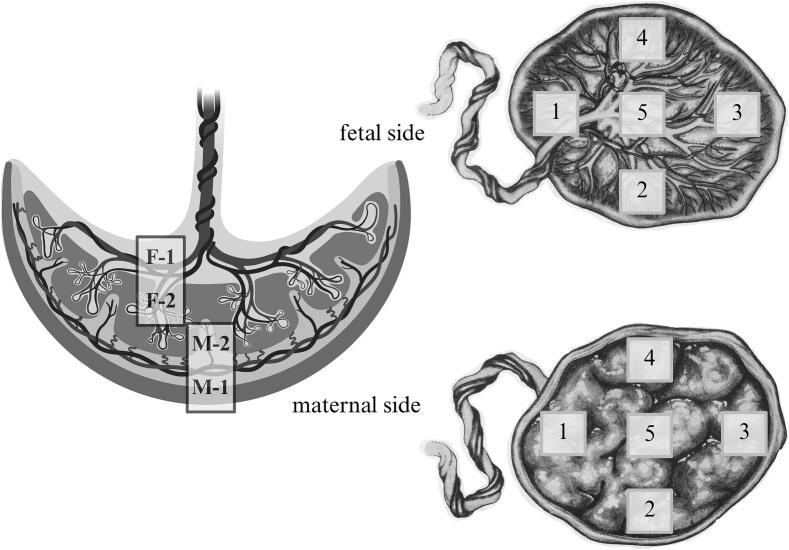
Placental sampling**,** created in BioRender.com F/M-1 – outer surface of a placenta from the fetal/maternal side. F/M-2 – inner part of a placenta from the fetal/maternal side. Numbers 1, 2, 3 and 4 are samples from the edge of the placenta, and number 5 is a sample from the central part of the placenta.

After termination, tissue samples were collected to investigate the discordance between NIPT and diagnostic results. Ten biopsies were taken from five areas on both the fetal and maternal sides of the placenta (Fig. 1). Eight placental samples underwent confirmatory testing using a modified NIPT protocol tailored for tissue. Finally, three fetal tissue samples (skin, adipose, and muscle tissue) were analysed to confirm the non-mosaic status previously indicated by amniocentesis.

In placental tissues, T18 was detected in a mosaic pattern with substantial variability in mosaicism levels across biopsies (Table 1). Higher levels of T18 mosaicism were observed on the fetal side of the placenta, with a maximum of 76% in the central region, while lower levels were detected on the maternal side (20%). At the placental edge, fetal-side mosaicism ranged between 40% and 53%, and maternal-side levels ranged between 30% and 33%. The fetus was non-mosaic, with chromosomal T18 detected in 97–99% of cells across all examined tissues.

**Table 1:** Determined levels of mosaicism in different parts of the placenta and fetus.

Source	Sample	Level of mosaicism
Central part of the placenta	Fetal side – outer surface, F-1-5 (amnion, chorionic plate)	76%
	Fetal side – inner part, F-2-5 (stem villi)	29%
	Maternal side – inner part, M-2-5 (floating villi)	27%
	Maternal side – surface, M-1-5 (anchoring villi, floating villi, decidua infiltrated with trophoblast)	20%
Edge of the placenta	Fetal side – outer surface, F-1-1 (amnion, chorionic plate)	40%
	Fetal side – inner part, F-2-1 (stem villi)	53%
	Maternal side – inner part, M-2-1 (floating villi)	33%
	Maternal side – surface, M-1-1 (anchoring villi, floating villi, decidua infiltrated with trophoblast)	30%
Fetal tissue	Skin	99%
	Adipose tissue	97%
	Muscle tissue	99%

## 3 Discussion

This case demonstrates a rare but clinically important false-negative NIPT result for T18 in a healthy pregnant woman carrying a non-mosaic trisomic fetus, despite adequate overall fetal fractions (9.66%, 7.64%, and 10.66%) and uninformative and negative screening results from NIPT, but abnormal first-trimester ultrasound findings (increased nuchal translucency, tricuspid regurgitation, and hydrops fetalis). Although the sensitivity of NIPT for T18 is reported to exceed 97% [4], the present case emphasizes the biological limitations of the test, particularly in the context of feto-placental mosaicism. All three non-invasive prenatal tests (including one after confirmed fetal diagnosis) failed to detect the aneuploidy, and the likely explanation lies in the spatial distribution of the mosaic cell lines within the placenta.

Given the potential for both false-positive and false-negative results in NIPT, especially in cases presenting with abnormal ultrasound findings, definitive diagnosis should be established through an invasive test [3]. In this case, due to the presence of multiple sonographic anomalies suggestive of a chromosomal abnormality, amniocentesis was performed, and the diagnosis of T18 was confirmed. These findings underscore the importance of integrating ultrasound screening with NIPT, as relying solely on the NIPT result could have delayed invasive testing and potentially led to a misdiagnosis.

Based on the results from amniocentesis, the pregnancy was terminated, and a post-termination analysis was performed.

Given the potential for uneven distribution of chromosomal abnormalities within the placenta, sampling multiple regions is crucial when placental mosaicism is suspected. Collecting and analysing biopsies from both the fetal and maternal sides of the placenta improve the reliability of confirmatory testing of chromosomal abnormalities in cases of discordant NIPT results [10].

Post-termination analysis of the placenta revealed uneven distribution of T18 mosaicism levels, with substantially higher levels on the fetal side (up to 76%) than on the maternal side (20–30%) of the placenta (Table 1). Given that cffDNA used for NIPT originates predominantly from the maternal side, this pattern markedly reduced the proportion of T18-positive DNA fragments in maternal plasma, significantly limiting the performance of NIPT and contributed to the false-negative results. The estimated “effective fetal fractions” for T18 (1.5%–3.1%) were below the current NIPT detection threshold (∼4%). This mechanism is consistent with findings by Gao et al. [11], who showed that the combination of low placental mosaicism levels on the maternal side and modest total fetal fraction can result in trisomy-specific fractions insufficient for detection.

The current findings also support previous reports that feto-placental mosaicism is responsible for a proportion of false-negative NIPT results in approximately 1 in 64 T18 cases [2]. Analysis of unpublished prospective data from the Trisomy test® in 20,288 pregnancies indicates a comparable rate, with approximately 1 in 48 T18 cases missed due to this mechanism. Investigation of discrepancies enhances understanding of NIPT limitations and contributes to improving its accuracy in clinical use.

## 4 Conclusion

This case highlights the limitations of NIPT in detecting chromosomal abnormalities when feto-placental mosaicism is present. Despite high sensitivity and specificity, NIPT screening can yield false-negative results, particularly in rare cases of feto-placental mosaicism. Pretest counselling should emphasize the origin and limitations of cffDNA to ensure informed decision-making and appropriate follow-up in prenatal care. NIPT is currently the best screening tool in combination with the first-trimester combined test for prenatal screening of aneuploidies, but if sonographic abnormalities are identified, diagnostic testing relying on invasive methods to gain relevant biological material of fetal origin remains essential.

## Contributors

Natália Andová contributed to conception of the case report, interpreting the data, drafting the manuscript and revising the article critically for important intellectual content.

Vanda Repiská contributed to conception of the case report and revising the article critically for important intellectual content.

Petra Priščáková contributed to interpreting the data and drafting the manuscript.

Lajos Gergely contributed to interpreting the data and revising the article critically for important intellectual content.

Michaela Hýblová contributed to patient care and acquiring and interpreting the data.

Gabriel Minárik contributed to patient care, conception of the case report, acquiring the data and revising the article critically for important intellectual content.

All authors approved the final submitted manuscript.

## Patient consent

This case report is part of the “PrenaScreen” biomedical research project, which received approval on March 25, 2020, from the Ethics Committee in Bratislava (05006/2020/HF/2), and was conducted in accordance with the Declaration of Helsinki. Written informed consent was obtained from the patient for participation and for publication of this case report and accompanying images.

## Provenance and peer review

This article was not commissioned and was peer reviewed.

## Funding

This work was supported by the 10.13039/501100000780EU NextGenerationEU through the Recovery and Resilience Plan for Slovakia under the project No. 09I03-03-V0400253 and by the Ministry of Education, Research, Development, and Youth of the Slovak Republic through grant VEGA 1/0198/24.

## Declaration of competing interest

The authors declare the following financial interests/personal relationships which may be considered as potential competing interests:

Petra Priscakova reports financial support was provided by EU NextGenerationEU under the project No. 09I03-03-V04-00253. Vanda Repiska reports financial support was provided by the Ministry of Education, Research, Development, and Youth of the Slovak Republic through grant VEGA 1/0198/24. If there are other authors, they declare that they have no known competing financial interests or personal relationships that could have appeared to influence the work reported in this paper.
